# Supplementary material for: A prophage tail-like protein is deployed by Burkholderia bacteria to feed on fungi
Source: Nat Commun. 2017 Sep 1;8:404. doi: 10.1038/s41467-017-00529-0 (PMC5581363; doi:10.1038/s41467-017-00529-0)
Supplement: Supplementary file 1 — Supplementary Information [file 41467_2017_529_MOESM1_ESM.pdf]

Description of Supplementary Files

File Name: Supplementary Information
Description: Supplementary Tables and Supplementary Figures

File Name: Peer Review File

Supplementary Table 1: *B. gladioli* strain NGJ1 prevents formation of *R. solani* sclerotia, during confrontation

	No of sclerotia formed on PDA plates[a]				
	**Repeat 1**	**Repeat 2**	**Repeat 3**	**Repeat 4**	**Repeat 5**
R. solani	**190**	**170**	**185**	**175**	**198**
***R. solani* + NGJ1**[b]	**1**	**4**	**10**	**9**	**5**

[a]; No. of sclerotia formed on PDA plates after 7 days of confrontation with NGJ1

[b]; Sclerotia collected from the NGJ1 confrontation plates failed to germinate when grown on fresh PDA plate

Supplementary Table 2: *B. gladioli* strain NGJ1 treatment prevents germination of *R. solani* sclerotia

	Repeat 1[a]	Repeat 2[a]	Repeat 3[a]
R. solani	**5/5**	**5/5**	**5/5**
***R. solani* + heat killed NGJ1 (10^9 cells/ml) culture**	**5/5**	**5/5**	**5/5**
***R. solani* + 10^9 cells/ml NGJ1 culture**	**0/5**	**0/5**	**0/5**
***R. solani* +10^7 cells/ml NGJ1 culture**	**0/5**	**0/5**	**0/5**
R. solani* + 10^5 cells/ml NGJ1 culture	**2/5**	**3/5**	**1/5**
R. solani* +10^3 cells/ml NGJ1 culture	**5/5**	**5/5**	**5/5**

[a:] no of germinating/total no. of treated *R. solani* sclerotia on PDA plate

*: Although a few bacterial treated sclerotia could germinate initially but subsequently NGJ1 grew over them and prevented their growth

Supplementary Table 3: List of fungi used in this study

Sr. no.	Fungal strain	Growth temperature (°C)	Media	Source
**1**	*Rhizoctonia solani* strain BRS1	28	PDA	Lab collection
**2**	*R. solani* strain BRS8	28	PDA	Lab collection
**3**	*R. solani* strain BRS9	28	PDA	Lab collection
**4**	*Alternaria brassicae*	28	PDA	ITCC (IARI)
**5**	*Magnaporthe oryzae*	22	PDA	Lab collection
**6**	*Venturia inaequalis*	22	PDA	Lab collection
**7**	*Fusarium oxysporum* 549	28	PDA	Lab collection
**8**	*F. oxysporum* 7063	28	PDA	Lab collection
**9**	*Dedymella* sp.	28	PDA	Lab collection
**10**	*Phytophthora* sp.	25	PDA	ITCC (IARI)
**11**	*Colletotrichum* sp.	28	PDA	Lab collection
**12**	*Ascochyta rabiei*	22	PDA	Lab collection
**13**	*Neofusicoccum* sp.	28	PDA	Lab collection
**14**	*Alternaria sp.*	28	PDA	Lab collection
**15**	*Saccharomyces cerevisiae*	28	YPDA	Lab collection
**16**	*Candida albicans*	37	YPDA	Lab collection

Supplementary Table 4: List of bacterial strains, plasmids and primers

Strains or plasmid	Relevant characteristics	Source
***E. coli* strains**		
DH5α	F', endA1 hsdR17 (rk- mk+) supE44 thi-1 recA1 gyrA TelA1 cp8OdlacZAM15 A (lacZY A-argF) U169	Lab collection
S17-1	RP4-2-Tc::Mu-Km::Tnl pro hsdRrecA	Lab collection
BL-21	DE3-Codon+	Lab collection
***B. gladioli* strains**		
NGJ-1	Natural isolate	Lab collection
NGJ-100	*Bg_9562*::pGD2 rif-2; Bg_9562$^-$, Km^r derivative of NGJ-2	Current study
NGJ-101	*Bg_9562*::pGD2 rif-2; Bg_9562$^-$, Km^r derivative of NGJ-2	Current study
NGJ-2	*rif*-2, Rf^r derivative of NGJ-1	Current study
NGJ-102	NGJ-100/pGD3	Current study
NGJ-103	NGJ-101/pGD3	Current study
NGJ-12	*HrcC*::pGD4 rif-2; T3S$^-$, Km^r HR$^-$ derivative of NGJ-2	Current study
NGJ-13	*HrcC*::pGD4 rif-2; T3S$^-$, Km^r HR$^-$ derivative of NGJ-2	Current study
***R. solanacearum* strains**		
F1C1	Natural isolate	Lab collection
F1C1N2	*HrpB*::ΩSpc T3S$^-$, Sp^r, HR$^-$ derivative of F1C1	Lab collection
F1C1N3	F1C1/pGD3	Current study
F1C1N4	F1C1N2/pGD3	Current study
Pantoea ananatis	Natural isolate	Lab collection
**Plasmids**		
pET28a	Kanamycin, 5.320kb, colE1	Lab collection
PK18mob	pUC18 derivative; Mob+ Tra– Km^r	Lab collection
pGD1	pET28a::*Bg_9562*	Current study
pGD2	pK18mob::*Bg_9562* partial	Current study
pHM1	Broad-host-range cosmid vector (13.3 kb); Sp^r	Lab collection
pGD3	pHM1::*Bg_9562*	Current study
pGD4	pK18mob::*hrcC* partial	Current study
**List of primers**		
Bg_*9562* f F1	5′ CATATGAACACGGAAAACCAGGAT 3′	Current study
Bg_*9562* f R1	5′ AAGCTTCGCGCTCGGGGATTCCATGCT 3′	Current study
Bg_*9562* p F 2	5′ GAATTCATGCCGGCGCGCTGCGCGGC 3′	Current study
Bg_*9562* p R 2	5′ AAGCTTGCTCGGGGATTCCATGCTCGCT 3′	Current study
Bg_*9562* cf F3	5′ AAGCTTAACACGGAAAACCAGGAT 3′	Current study
Bg_*9562* cf R3	5′ GAATTCCGCGCTCGGGGATTCCATGCT 3′	Current study
M13 fwd	5’ TGTAAAACGACGGCCAGT 3’	Current study
M13 rev	5’ AGGAAACAGCTATGACCAT 3’	Current study
hrcCF	5’ GAATTCATGTACAGCGTGAAGTCGGG 3’	Current study
hrcCR	5’ AAGCCTATCACCGGAATGTCGTCCAC 3’	Current study
Hrccf1	5’ ATG AGAGCGAAGAAGCTTGTTGC	Current study

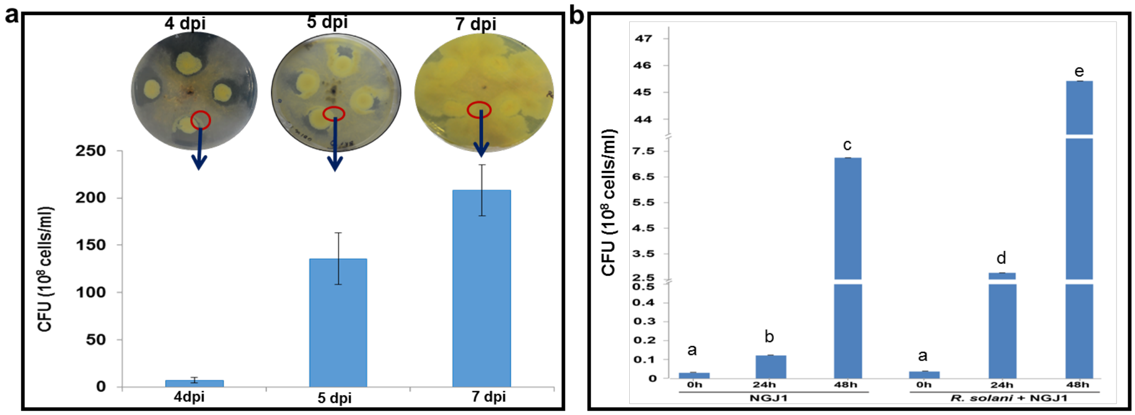

Supplementary Figure 1. Presence of *R. solani* mycelia promotes the growth of *B. gladioli* strain NGJ1. a, The NGJ1 was found growing over *R. solani* mycelia during confrontation on PDA plates. The bacterial abundance on fungal mycelia was estimated at different time points (4, 5 and 7 dpi) of confrontation and data is presented as bar chart. The photographs in the inset represent the progression of mycophagy on *R. solani*. The red circle reflects the sampling site used for bacterial counting. **b,** Bacterial growth in minimal media (CDB) with and without presence of *R. solani* mycelia at different time points. NGJ1 growth was drastically enhanced in presence of fungal mycelia. The experiments were independently repeated three times with minimum three technical replicates. Values with different letters are significantly different at $P<0.001$ (estimated using one-way ANOVA). Graphs show mean values ± standard deviation.

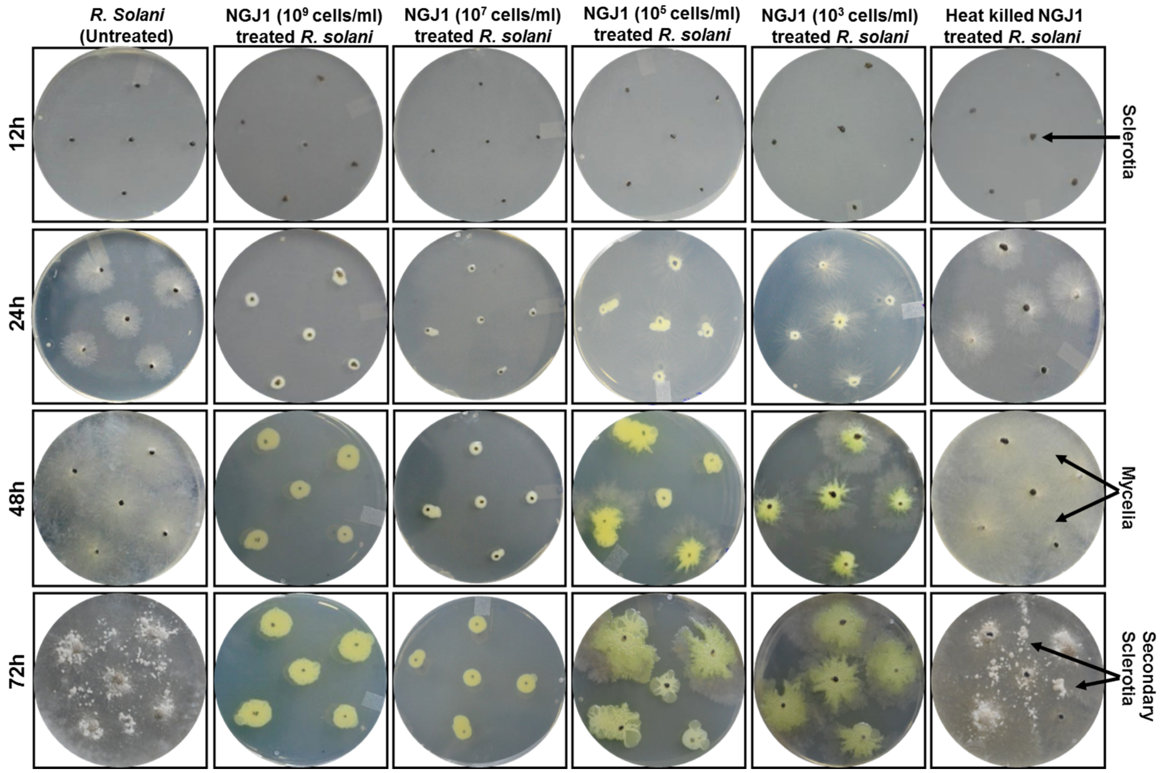

Supplementary Figure 2. Effect of different concentrations of *B.* gladioli strain NGJ1 treatment on *R. solani* sclerotia. 10^9 or 10^7 cells/ml NGJ1 treated *R. solani* sclerotia failed to germinate when placed on fresh PDA plates. Upon treatment with 10^5 or 10^3 cells/ml bacterial cultures, the sclerotia could initially germinate. But subsequently bacteria grew over fungal biomass and inhibited their further growth. However, the sclerotia that were either not treated with bacteria or treated with heat killed bacteria showed proper growth on PDA plates. Similar results were obtained in at least three independent biological experiments and only representative photographs are shown.

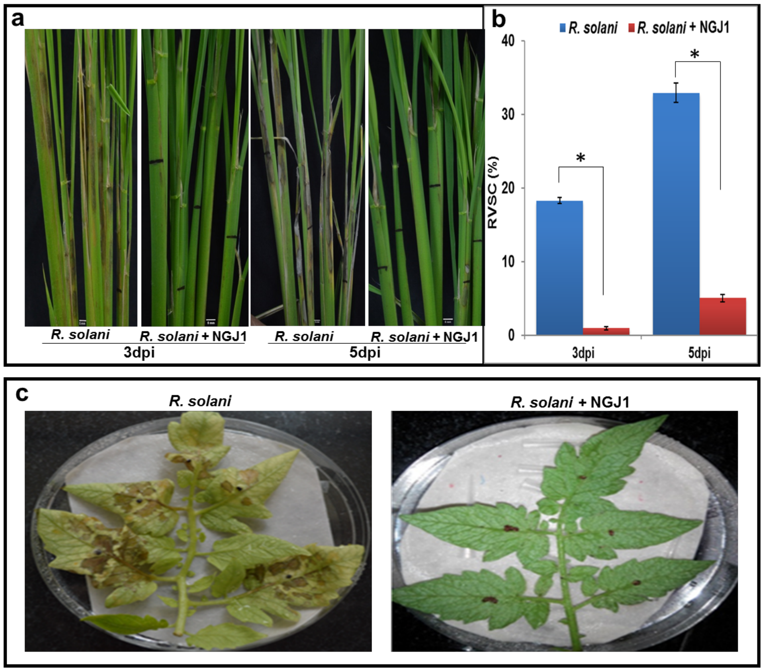

Supplementary Figure 3. *B. gladioli* strain NGJ1 treatment suppresses disease causing ability of *R. solani*. a, Representative images of rice sheath showing disease symptoms caused by NGJ1 (10^9 cells/ml) treated or buffer (10mM PBS, pH: 7.0) treated *R. solani* sclerotia, at 3 dpi and 5 dpi. **b**, Disease index of infected rice sheath (cv. TP309) with bacteria or buffer treated *R. solani* sclerotia, in terms of relative vertical sheath colonization (RVSC). **c,** Representative images of tomato (cv. pusa ruby) leaves infected with *R. solani* sclerotia with or without bacterial treatment, at 3 dpi. The experiments were repeated three times and each time 15 tillers/leaves were analyzed. Asterisks indicate significant difference between indicated groups at $P<0.001$, estimated by one-way ANOVA. Graphs show mean values ± standard deviation.

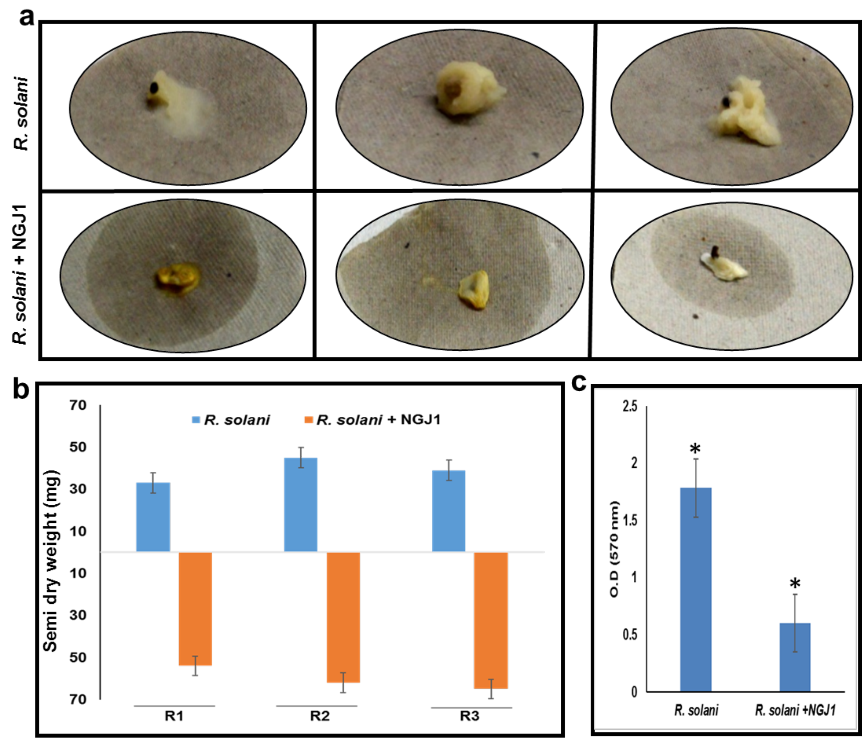

Supplementary Figure 4. Effect of *B. gladioli* strain NGJ1 treatment on fungal biomass. a, Observed *R. solani* biomass, with or without NGJ1 treatment after 48h of growth in PDB media**. b,** Graph representing reduction in fungal biomass upon NGJ1 treatment and increase in biomass in case of untreated *R. solani* mycelia, after 48h of growth in PDB media. R1, R2 and R3 represent data from 3 independent biological replicates. **c,** Cell viability estimated by MTT staining of NGJ1 treated and untreated mycelia. The experiments were repeated with three technical as well as biological replicates. Asterisks indicate statistical significant difference between indicated groups at $P<0.001$ (estimated by one-way ANOVA). Graphs show mean values ± standard deviation.

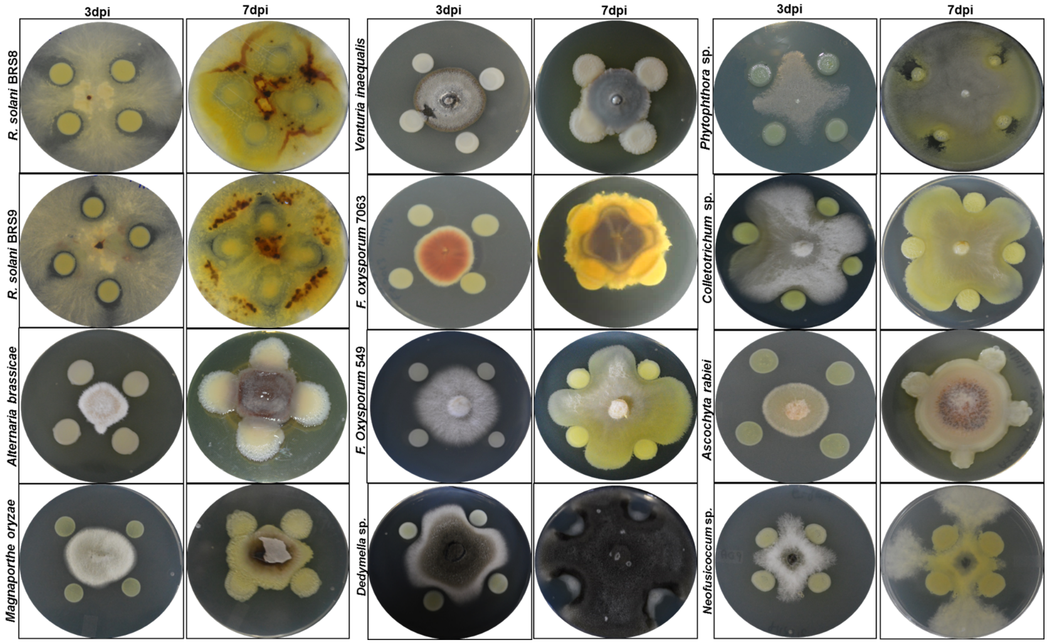

Supplementary Figure 5. *B. gladioli* strain NGJ1 demonstrates mycophagy on various fungi. The mycophagous behavior of NGJ1 was observed on various fungi including *Rhizoctonia solani* strain BRS8, *R. solani* strain BRS9, *Alternaria brassicae*, *Magnaporthe oryzae*, *Venturia inaequalis, Fusarium oxysporum* strain *7063, F. oxysporum* strain *549,* Dydmella *sp*, *Phytophthora sp*, *Colletotricum sp, Ascochyta rabiei*, *Neofusicoccum sp*. Images of bacterial-fungal confrontation at 3 and 7 dpi are represented. Similar results were obtained in at least three independent biological experiments and only representative photographs are shown.

a. Genomic organization of T3SS encoding genes in *B. gladioli* NGJ1 genome

T3SS_cluster_type	Num_components	Core_Components in_cluster	Total_Core_Comp_ NFT3SS	Perc_core
Non-flagellar	21	10	9	100%

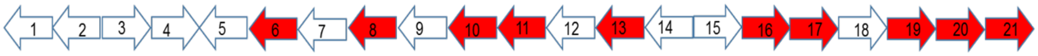

b. Locus IDs and name of T3SS encoding genes in *B. gladioli* NGJ1 genome

Gene no.	Gene	Locus I.D	Protein	Component	Accession No.
1	orf_3833	ACI79_RS25520	HrpK	None core	WP_047838853
2	orf_3834	ACI79_RS25525	PKHD type Hydroxylase	Unknown	WP_047838854
3	orf_3835	ACI79_RS25530	DUF3455 domain protein	Unknown	WP_047838855
4	orf_3836	ACI79_RS25535	HrpG	Unknown	WP_047838856
5	orf_3837	ACI79_RS25540	HrpW	Unknown	WP_047838857
6	orf_3838	ACI79_RS25545	HrcC	Core	WP_013689095
7	orf_3839	ACI79_RS25550	HrpB	Non core	WP_047839013
8	orf_3840	ACI79_RS25555	HrcT	Core	WP_047838858
9	orf_3841	ACI79_RS25560	HrpB7/HrpD	Non core	WP_047838859
10	orf_3842	ACI79_RS25565	HrcN	core	WP_036054803
11	orf_3843	ACI79_RS25570	HrcL	core	WP_047838860
12	orf_3844	ACI79_RS25575	HrpB4/HrpH	Non core	WP_053062820
13	orf_3845	ACI79_RS25580	HrcI/HrcJ	Core	WP_047838862
14	orf_3846	ACI79_RS25585	HrpB2/HrpJ	Non core	WP_047838863
15	orf_3847	ACI79_RS25590	HrpB1/HrpK	Non core	WP_013689086
16	orf_3848	ACI79_RS25595	HrcU	Core	WP_017921746
17	orf_3849	ACI79_RS25600	HrcV	Core	WP_047838864
18	orf_3850	ACI79_RS25605	HpaP	Unknown	WP_047838865
19	orf_3851	ACI79_RS25610	HrcQ	Core	WP_053062813
20	orf_3852	ACI79_RS25615	HrcR	Core	WP_013689081
21	orf_3853	ACI79_RS25620	HrcS	Core	WP_013689080

Supplementary Figure 6. *B. gladioli* strain NGJ1 harbors genes potentially encoding Type III secretion system. a, Genomic organization of T3SS encoding genes in *B. gladioli* NGJ1 genome. Out of 21 different genes, 10 were found to encode the core components of T3SS apparatus. There was 100% (9/9) conservation of core components that are associated with a functional non-flagellar type of T3SS apparatus (http://bacterial-virulence-factors.cbgp.upm.es/T346Hunter). **b**, The gene name, locus ID, encoded protein, predicted role in T3SS apparatus as well as corresponding accession number as per *Burkholderia* genome database (http://www.burkholderia.com) are mentioned.

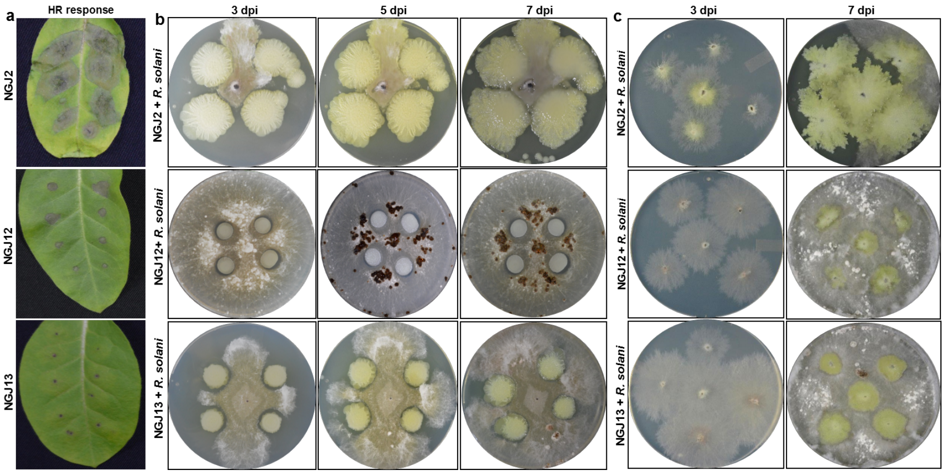

Supplementary Figure 7. T3SS deficient mutant strains of *B. gladioli* strain NGJ1 are defective in mycophagy. **a**, HR response in *Nicotiana benthamiana* leaves infiltrated with different *B. gladioli* strains. Both T3SS mutants (NGJ12 and NGJ13) were unable to elicit HR response, while the NGJ2 (rif^R derivative of NGJ1) elicited HR. **b**, The confrontation of T3SS mutants with *R. solani* on PDA plates. Both T3SS mutants failed to demonstrate mycophagy while NGJ2 exhibited mycophagy. **c**, The T3SS mutants were defective in mycophagy even at lower dilution. The *R. solani* sclerotia treated with 10^5 cells/ml culture of T3SS mutant bacteria failed to inhibit fungal growth and could not forage over growing mycelia. On the other hand, the NGJ2 (10^5 cells/ml) treatment prevented sclerotial growth and demonstrated mycophagy. Similar results were obtained in at least three independent experiments.

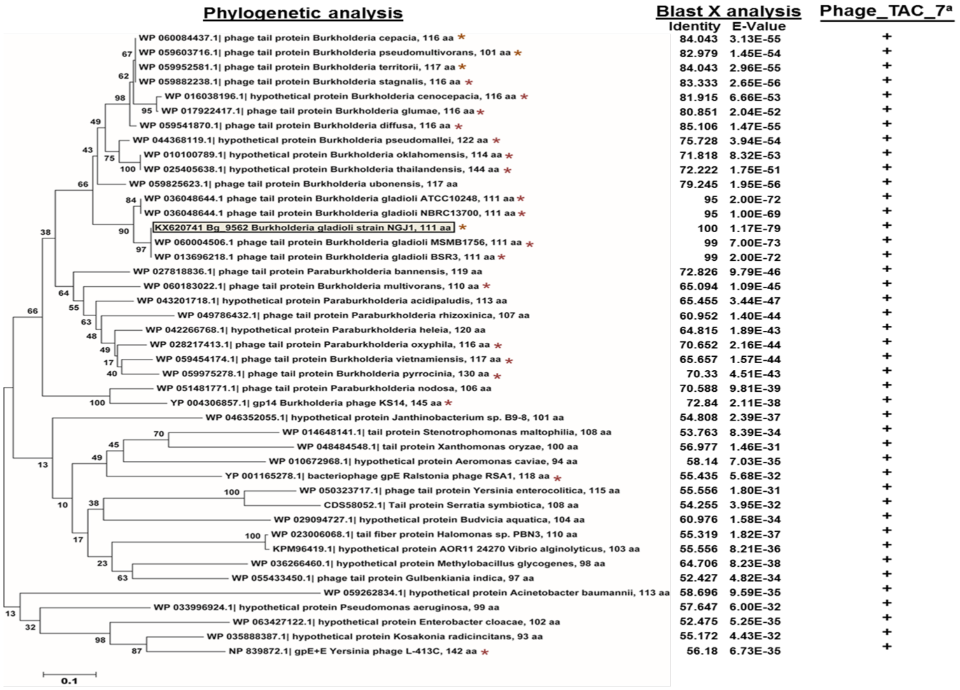

[a] CDD: NCBI conserved domain database was used to identify the conserved domain present in different orthologs
'+' sign indicates the presence of Phage_TAC_7 domain

Supplementary Figure 8. Phylogenetic and blastX analysis reflecting the similarity of Bg_9562 protein with other phage tail proteins. The evolutionary analysis of different phage tail proteins were conducted in MEGA6 and distances were computed using the JTT matrix-based method. The evolutionary tree was inferred using Neighbor-Joining method with bootstrap test (100 replicates). The Bg_9562 protein as well as its different orthologs were found to be of similar length (number indicates amino acid length). The Bg_9562 homologs that are located in apparent bacteriophage cluster in different bacteria are highlighted with asterisks. Percentage identity and the E-value of Bg_9562 protein with phage tail proteins of different bacterial strains are shown next to corresponding branches. Presence of conserved phage_*TAC_7* domain, generally present in bacteriophage phage tail protein Gp41, was detected in Bg_9562 protein as well as its various orthologs.

a. Genomic organization of *Bg_9562* gene in NGJ1 draft genome

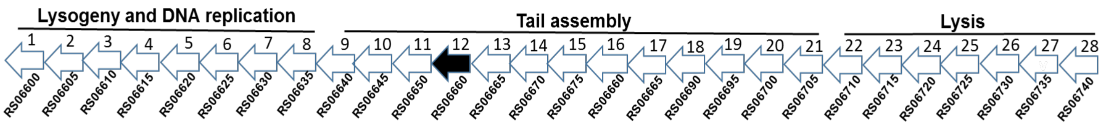

b. The *Bg_9562* gene homologs in different *B. gladioli* strains are located in bacteriophage cluster

Gene	NGJ1	ATCC 10248	NBRC13700	MSMB1756	BSR3
1	Integrase	Integrase	Integrase	Integrase	Integrase
2	Zing finger CHC2 type protein	Hypothetical protein	Zing finger CHC2 type protein	Hypothetical protein	Hypothetical protein
3	Hypothetical protein	Hypothetical protein	Hypothetical protein	Hypothetical protein	Hypothetical protein
4	Hypothetical protein	Hypothetical protein	Hypothetical protein	Transcriptional regulator	Zing finger CHC2 type protein
5	Transcriptional regulator	Transcriptional regulator	Transcriptional regulator	Hypothetical protein	Hypothetical protein
6	Hypothetical protein	Hypothetical protein	XRE transcriptional regulator	Phage late control protein	putative phage transcriptional activatior
7	XRE transcriptional regulator	XRE transcriptional regulator	Hypothetical protein	Oxidoreductase	Hypothetical protein
8	Hypothetical protein	Hypothetical protein	Phage late control protein	Hypothetical protein	Hypothetical protein
9	Phage late control protein	Phage late control protein	Oxidoreductase	Phage tail protein	Fels-2 prophage protein
10	Oxidoreductase	Oxidoreductase	Hypothetical protein	Major tail tube protein	Hypothetical protein
11	Hypothetical protein	Hypothetical protein	GpE family protein	Tail sheath protein	Phage related tail transmembrane protein
**12**	**Phage tail protein**	**Phage tail protein**	**Phage tail protein**	**Phage tail protein**	**Hypothetical protein**
13	Major tail tube protein	Major tail tube protein	Major tail tube protein	Phage tail protein	Phage tail protein
14	Tail tube protein	Tail tube protein	Tail tube protein	Tail protein	Major tail tube protein
15	Phage tail protein(Pseudogene)	Tail fibre assembly protein	Tail fibre assembly protein	Base plate assembly protein	Hypothetical protein
16	Phage tail collar domain protein	Phage tail collar domain protein	Phage tail collar domain protein	Base plate assembly protein	Bacteriophage acquired protein
17	Tail protein	Tail protein	Tail protein	Base plate assembly protein	Tail fibre protein
18	Base plate assembly protein	Base plate assembly protein	Base plate assembly protein	Fels-2 prophage protein	Tail fibre protein
19	Base plate assembly protein	Base plate assembly protein	Base plate assembly protein	LysC	Base plate assembly protein
20	Base plate assembly protein	Base plate assembly protein	Base plate assembly protein	LysB	Base plate assembly protein
21	Fels-2 prophage protein	Fels-2 prophage protein	Fels-2 prophage protein	Hypothetical protein	Hypothetical protein
22	LysC	LysC	LysC	Hypothetical protein	Fels-2 prophage protein
23	LysB	LysB	LysB	Membrane protein	LysC
24	Peptidoglycan binding protein	Hypothetical protein	Hypothetical protein	Tail protein	LysB
25	Phage encoded membrane protein	Phage encoded membrane protein	Phage encoded membrane protein	Hypothetical protein	Bacteriophage acquired protein
26	Phage encoded membrane protein	Phage encoded membrane protein	Phage encoded membrane protein	Hypothetical protein	Phage encoded membrane protein
27	Tail protein	Tail protein	Tail protein	Hypothetical protein	Phage encoded membrane protein
28	Hypothetical protein	Hypothetical protein	Hypothetical protein	Hypothetical protein	Phage tail protein X

Supplementary Figure 9. Homologues of *Bg_9562* are located in bacteriophage genomic cluster in different *B. gladioli* genomes. **a,** The genomic organization of *Bg_9562* gene (filled arrow) in *B. gladioli* strain NGJ1. The number reflects genes that are present along with *Bg_9562* and their corresponding locus IDs as per *Burkholderia* genome database are mentioned. The involvement of these genes in various phage related functions are underlined. **b,** The genomic organization of *Bg_9562* homologs in different *B. gladioli* strains. The putative functions of different genes present in that locus are tabulated. The bacteriophage locus seems incomplete in various *B. gladioli* strains as the genes encoding capsid/head assembly proteins are absent.

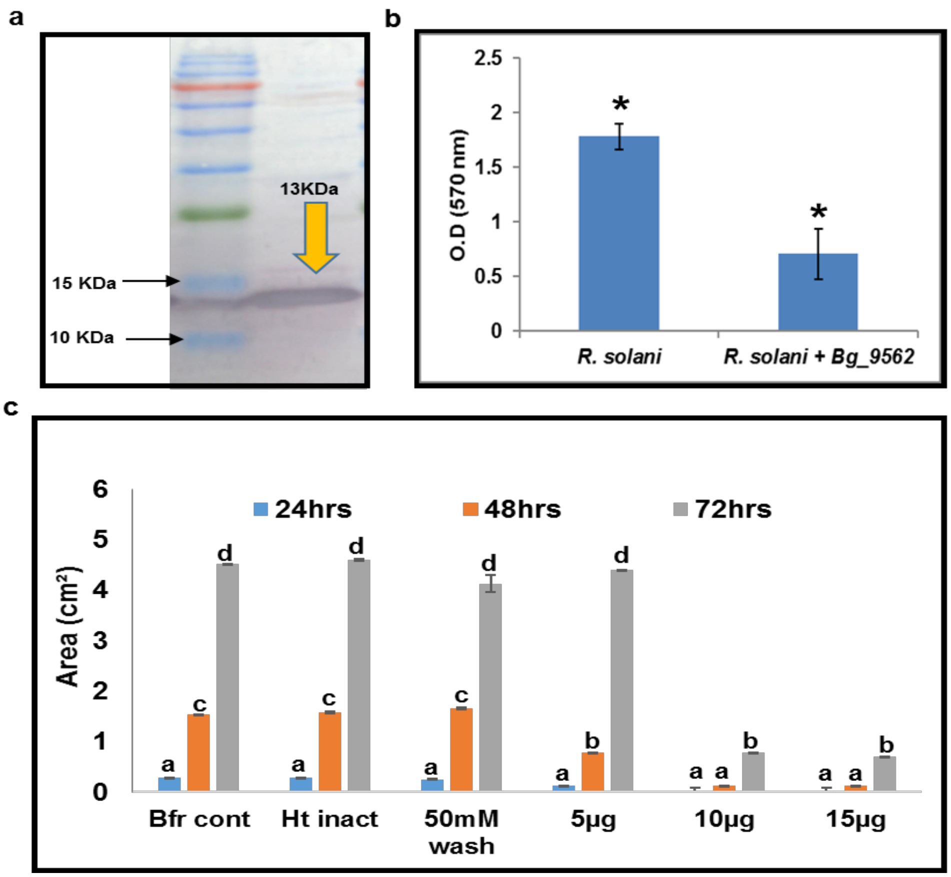

Supplementary Figure 10. Characterization of Bg_9562 protein. a, Western blot analysis of recombinant Bg_9562 protein purified from *E. coli*, using anti-His antibody. **b,** Cell viability estimated by MTT staining of Bg_9562 protein and buffer treated *R. solani* mycelia. **c,** Fungal growth (area of observed mycelial growth) inhibition observed upon protein treatment. 15 µg/ml of protein was efficient in preventing fungal growth while various control solutions did not affect mycelia growth. The experiments were repeated three times with three replicates. Asterisks and different letters indicate significant difference between indicated groups at *P*<0.001 and *P*<0.05 (estimated by one-way ANOVA), respectively. Graphs show mean values ± standard deviation.

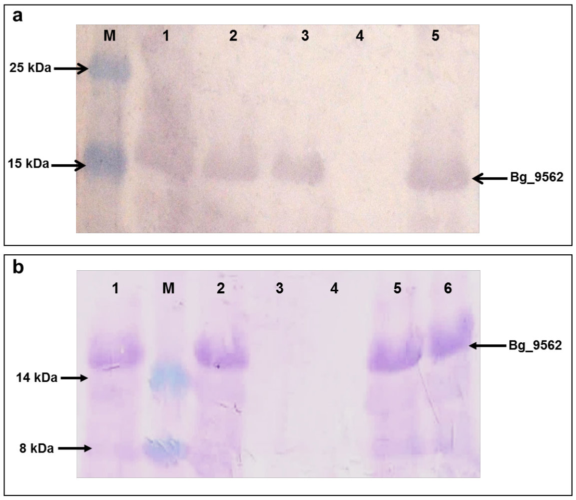

Supplementary Figure 11. Western blot analysis suggests that during mycophagous interaction NGJ1 translocates the Bg_9562 protein into *R. solani* using type III secretion system. Western blot analysis was performed using Bg_9562 protein specific polyclonal antibody. **a,** NGJ1 translocates Bg_9562 protein into *R. solani* mycelia. The protein isolated from various samples; NGJ1 pellet (Lane 1), NGJ1 treated mycelia (Lane 2 and 3), untreated mycelia (Lane 4), purified Bg_9562 protein (Lane 5) along pre-stained protein ladder (M) were blotted. **b,** T3SS mutant strains were defective in translocating the Bg_9562 protein into *R. solani* mycelia. The protein isolated from various samples; NGJ1 pellet (Lane 1), mycelia treated with NGJ1 (Lane 2), mycelia treated with T3SS mutants: NGJ12 (lane 3) and NGJ13 (Lane 4), bacterial pellet of NGJ12 (lane 5) and NGJ13 (lanes 6) along with pre-stained protein ladder (M) were blotted. The arrow indicates Bg_9562 specific band. Similar results were obtained in three independent biological replicates.

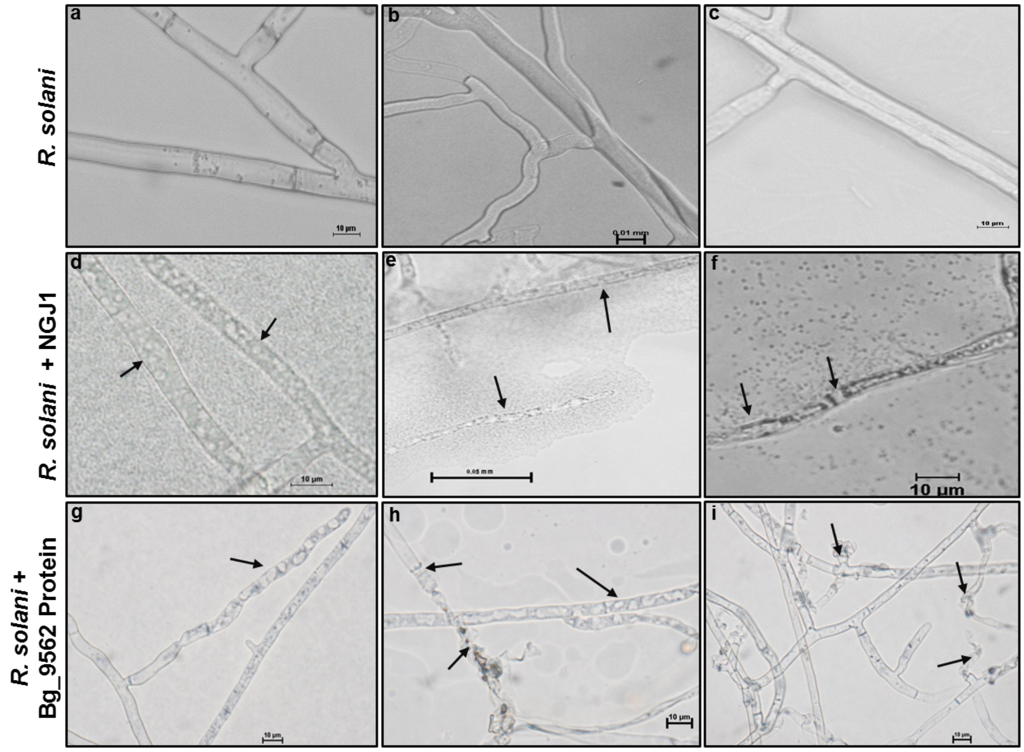

Supplementary Figure 12. Microscopic analysis of *B. gladioli* strain NGJ1 and Bg_9562 protein treated *R. solani* mycelia. a- c, Control (untreated) *R. solani* mycelia. **d- f,** Characteristic alterations imparted in *R. solani* mycelia upon co-cultivation with NGJ1**.** The bacteria caused various structural changes such as membrane disruption, cytoplasm squeezing, hyphal disintegration and release of cytoplasmic granules. **g- i,** Alterations in hyphal integrity, deflated and degenerating hypha etc. were imparted by Bg_9562 protein treatment. Interestingly alterations caused by protein or bacterial treatment in *R. solani* mycelia were found to be quite similar. Similar results were obtained in at least three independent biological experiments. Arrows highlight the mycelial damage as mentioned above.

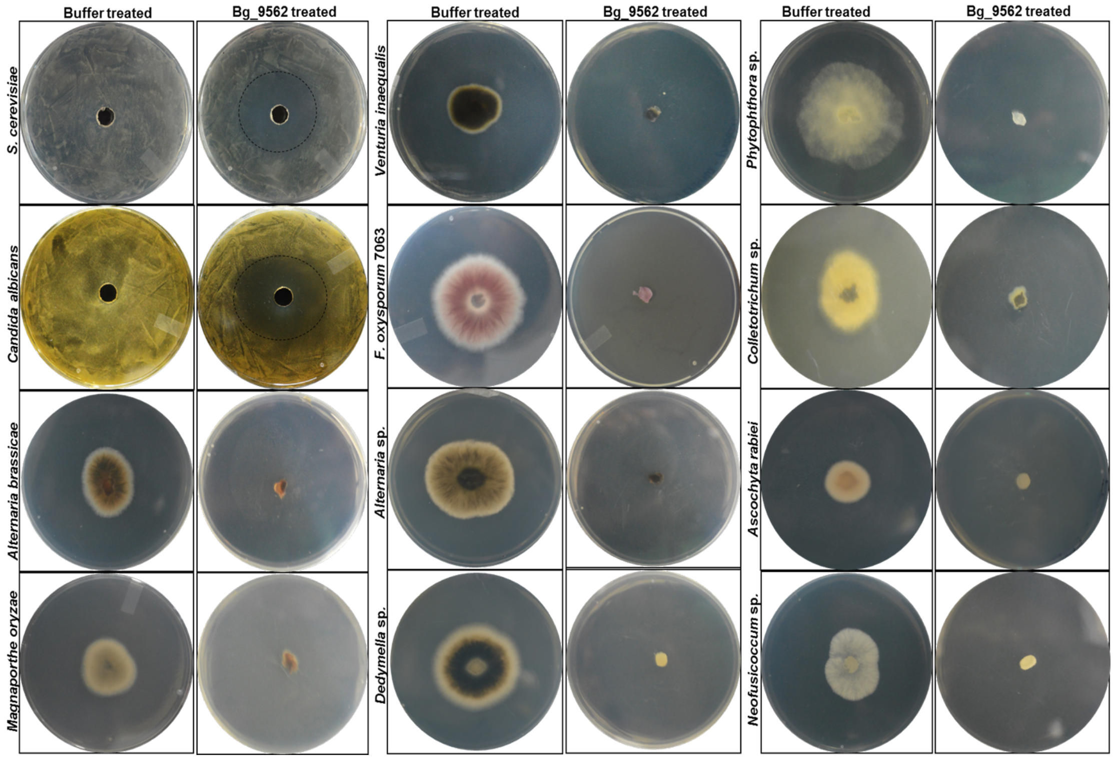

Supplementary Figure 13. Bg_9562 protein demonstrates broad spectrum anti-fungal activity. Bg_9562 protein shows strong antifungal activity on different fungi that includes *Saccharomyces cerevisiae, Candida albicans, Alternaria brassicae, Magnaporthe oryzae, Venturia inaequalis*, *Fusarium oxysporum* 7063, *Alternaria sp, Dydmella sp, Phytophthora sp, Colletotricum sp., Ascochyta rabiei, Neofusicoccum sp.* The protein treatment prevented growth of fungi while the buffer treated ones demonstrated optimum growth on PDA plates. For most of the fungi, data represents images of fungal growth at 24h post treatment. However, for slow growing fungi such as *V. inaequalis*, *M. oryzae* and *A. rabiei*, the data is presented at 48h of post treatment. Similar results were obtained in at least three independent biological repeats and only representative images are depicted.

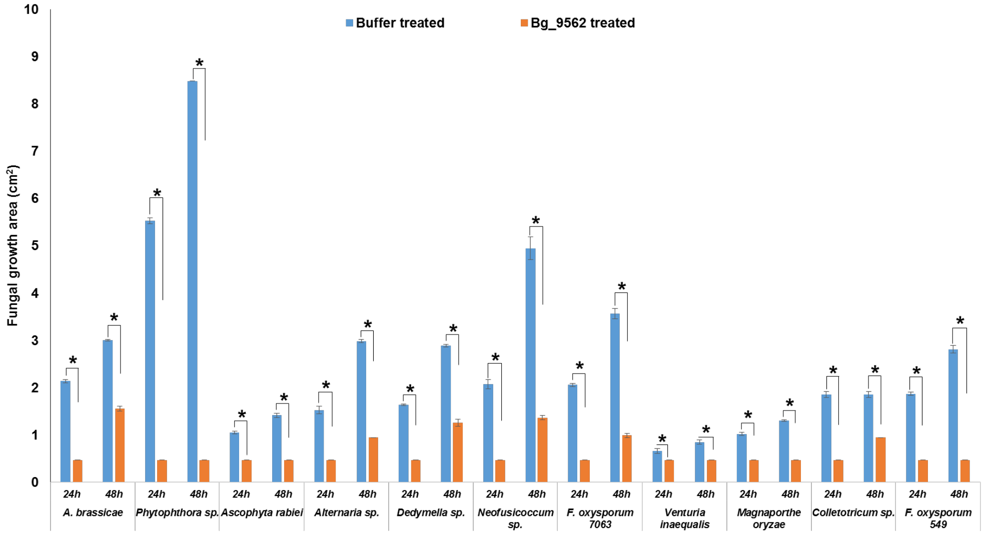

Supplementary Figure 14. Quantitative analysis of anti-fungal activity of Bg_9562 protein. The protein and buffer treated fungi were placed on PDA plates and their radial growth were estimated. The mycelial growth area observed for different fungi during 24h and 48h of growth post treatment is plotted as bar chart. The experiments were repeated with three technical and biological replicates. Asterisks indicate significant differences between indicated groups at $P<0.001$ (estimated by one-way ANOVA). Graphs show mean values ± standard deviation.

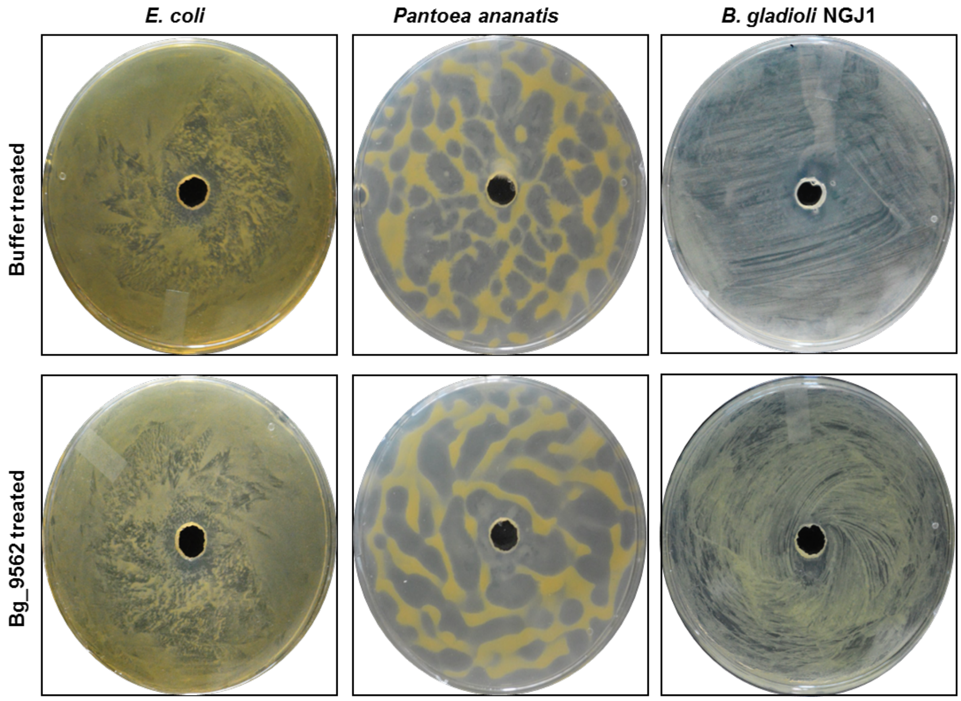

Supplementary Figure 15. Bg_9562 protein does not demonstrate anti-bacterial activity. The Bg_9562 protein did not inhibit the growth of different bacteria (*B. gladioli, P. ananatis* and *E. coli*). The bacterial growth pattern was found to be similar to that observed in case of buffer treated ones. Similar results were obtained in at least three independent experiments and only representative images are depicted.

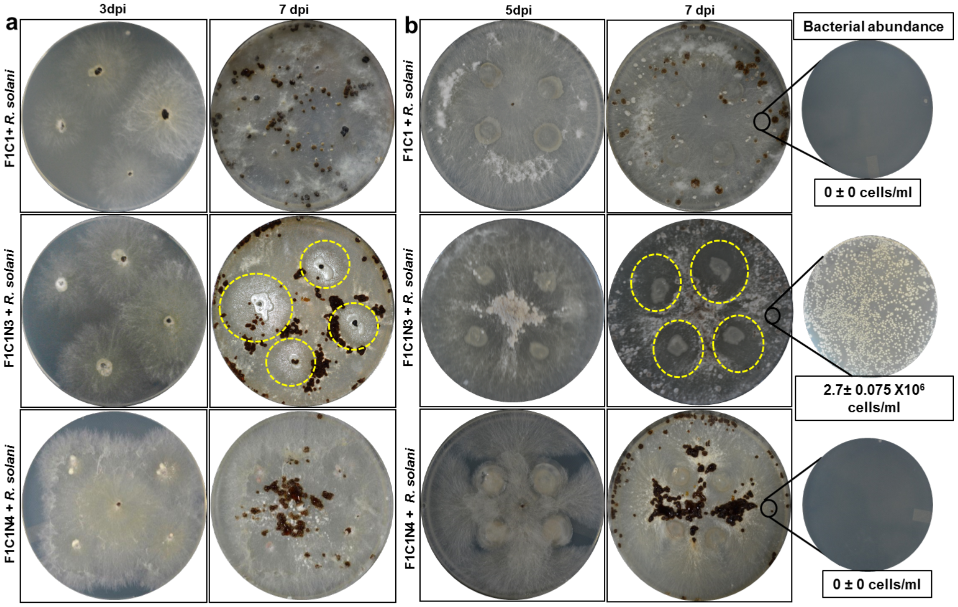

Supplementary Figure 16. ***Ralstonia solanacearum*** **strain F1C1 expressing** ***Bg_9562*** **gene demonstrates mycophagy on** ***R. solani*** **in a functional T3SS dependent manner. a**, The *R. solani* sclerotia treated with different strains of *R. solanacearum* (F1C1: wild type, F1C1N3: F1C1 expressing *Bg_9562*, F1C1N4: T3SS$^-$ deficient F1C1 expressing *Bg_9562*) were placed on PDA plates for mycophagous development. The F1C1 and F1C1N4 failed to show mycophagy, while the F1C1N3 demonstrated mycophagous development and spread over fungal mycelia. **b**, Confrontation assay of *R. solani* with different strains of *R. solanacearum*. Upon confrontation, the F1C1N3 strain was found foraging over fungal mycelia while F1C1N4 as well as the F1C1 failed to grow over fungi. The mycophagous zones are highlighted in yellow dotted circles. Further the bacterial abundance on fungal mycelia was estimated by dilution plating on BG-agar plates (the photographs of the plates are shown in inset and corresponding mean ± S.D of cfu numbers are mentioned) which suggested that *Bg_9562* expressing *R. solanacearum* demonstrates mycophagous ability in a T3SS dependent manner. The experiments were repeated with three technical as well as biological replicates and the representative images are shown.

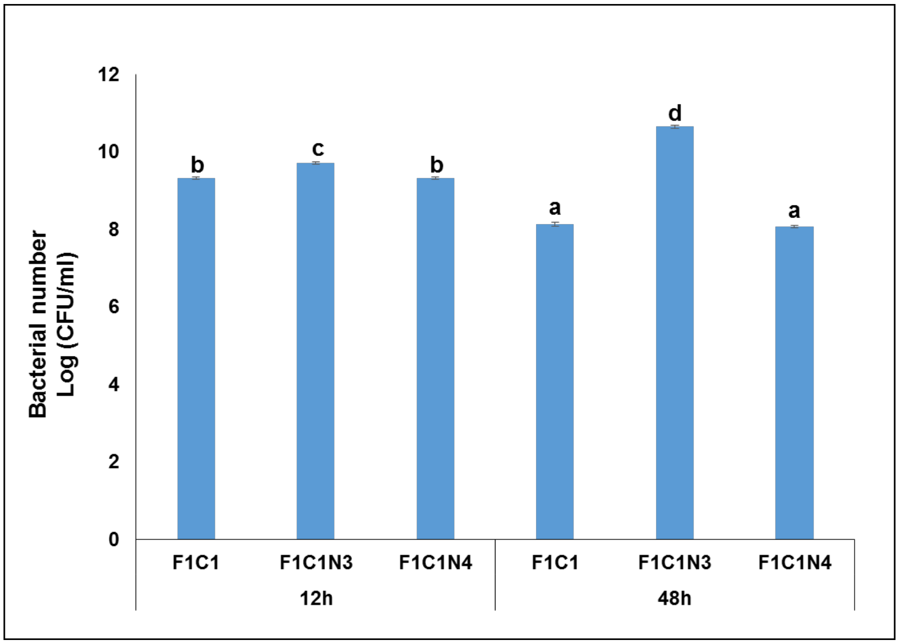

Supplementary Figure 17. Growth of *R. solanacearum* expressing Bg_9562 gene is enhanced in presence of *R. solani* mycelia. The growth rate of different *R. solanacearum* (F1C1, F1C1N3 and F1C1N4) strains were monitored after 24h and 48h of co-cultivation with *R. solani* mycelia in PDB broth by dilution plating on BG-agar plates and colony counting. The graph shows that presence of *R. solani* mycelium stimulated the growth of F1C1N3 in PDB media while the F1C1 as well as F1C1N4 strains failed to grow in presence of *R. solani*. The experiment was repeated with three technical and biological replicates. Values with different letters are significantly different at $P<0.001$ (estimated by one-way ANOVA). Graphs show mean values ± standard deviation.
